# Association between lifestyle factors and plasma adiponectin levels in Japanese men

**DOI:** 10.1186/1476-511X-4-27

**Published:** 2005-11-02

**Authors:** Rumi Tsukinoki, Kanehisa Morimoto, Kunio Nakayama

**Affiliations:** 1 Department of Social and Environmental Medicine, Osaka University School of Medicine, 2-2 Yamada-oka, Suita, Osaka 565-0871, JAPAN

**Keywords:** adiponectin, smoking, dietary factor, physical exercise, general Japanese men

## Abstract

**Background:**

Adiponectin is an adipocyte-specific protein that plays a role in obesity, insulin resistant, lipid metabolism, and anti-inflammation. Hypoadiponectinemia may be associated with a higher risk for type 2 diabetes and cardiovascular disease. Some studies suggest that adiponectin levels are modulated by lifestyle factors, but little is known about the associations between lifestyle factors and plasma adiponectin levels in Japanese people. We therefore investigated the associations between lifestyle factors and plasma adiponectin levels in general Japanese men.

**Methods:**

The subjects were 202 Japanese male workers who participated in an annual health check. They provided details about anthropometrical data, blood collection, their use of prescribed medication, and the clinical history of their families. They also completed a self-administered questionnaire about their lifestyles.

**Results:**

Subjects with plasma adiponectin levels below 4.0 μg/ml had significantly lower levels of HDL cholesterol and higher levels of BMI, SBP, DBP, total cholesterol, FBG, and platelets than did subjects with higher adiponectin levels. In multiple logistic regression after multiple adjustment, a plasma adiponectin level below 4.0 μg/ml was significantly associated with smoking (odds ratio [OR] = 2.08, 95% confidence interval [CI] = 1.01–4.30), a daily diet rich in deep-yellow vegetables (OR = 0.25, 95% CI= 0.07–0.91), frequent eating out (OR = 2.45, 95% CI = 1.19–5.08), and physical exercise two or more times a week (OR = 0.21, 95% CI = 0.06–0.74).

**Conclusion:**

Our findings show that adiponectin levels in general Japanese men are independently related to smoking, dietary factors, and physical exercise. We think that lifestyle habits might independently modulate adiponectin levels and that adiponectin might be the useful biomarker helping people to avoid developing type 2 diabetes and cardiovascular disease by modifying their lifestyles.

## Background

Adiponectin, an adipocyte-specific protein and one of the adipocytekines, is a 244-amino acid peptide with a structure highly homologous to complement factor C1q, collagen VIII, and collagen X [1,2]. Identified in the human adipose tissue cDNA library, it is encoded by adipose most abundant gene transcript 1 (apM1) [1] and is found in high concentrations in the peripheral circulation [2]. Adiponectin expression reflects peroxisome proliferators-activated receptor γ (PPAR-γ) [3,4], and is associated with the expression of tumor necrosis factor-α (TNF-α) [5]. Adiponectin expression is reduced in obesity individuals [2], and it is associated with lipid metabolism [6-8]. It modulates insulin action and resistance [9,10], and low adiponectin levels predict the development of type 2 diabetes [7,11-13]. And adiponectin play role in anti-inflammatory factor, and it is also related to the development of atherosclosis, hypertension, and coronary heart disease [14-17], and some reports show that adiponectin levels are associated with the inflammatory factors C-reactive protein (CRP), TNF-α, interleukin-6 (IL-6) and fibrinogen [18-20]. Male Japanese patients with hypoadiponectinemia (<4.0 μg/ml) show a significant 2-fold increase in the prevalence of coronary artery disease (CAD), independent of well-known CAD risk factors [14], and adiponectin levels below 4 μg/ml are closely associated with the clinical phenotype of the metabolic syndrome in Japanese men [21].

Although cross-sectional studies and studies in weigh-loss programs suggest that adiponectin levels are modulated by lifestyle factors such as nutritional variables, moderate alcohol intake, and smoking [22-27], little is known about the associations between lifestyle factors and plasma adiponectin levels in Japanese [27]. Iwashima et al. have shown in a study of 98 healthy Japanese men and 233 Japanese men with hypertension, diabetes, and hyperlipidemia that adiponectin levels are associated with habitual smoking [27]. Therefore, we investigated cross-sectionally the associations between lifestyle factors and plasma adiponectin levels in general Japanese men.

## Methods

### Subjects

**Figure 1:**
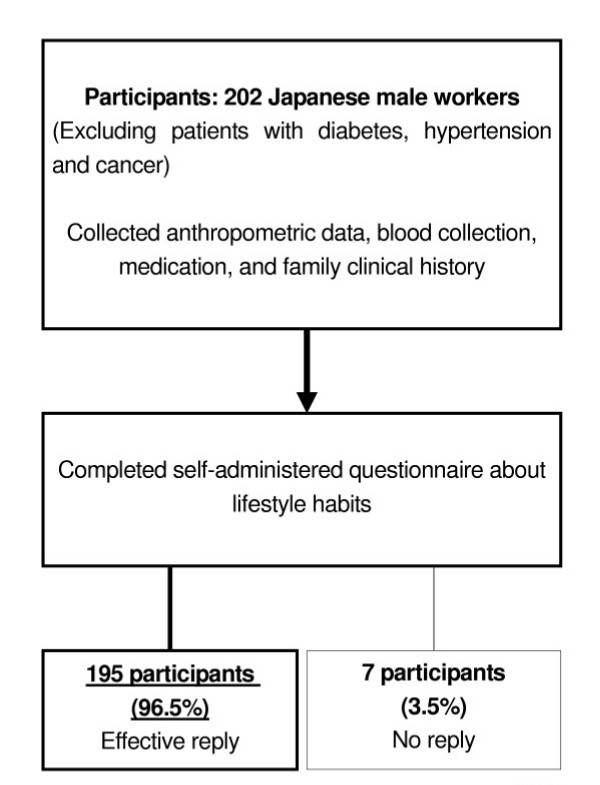
A flow-chart of the sampling procedure for 202 Japanese male factory workers.

The subjects were 202 Japanese male workers at a metal plant who were participating in an annual health check for employees in October 2003 (Figure 1). Anthropometric dates and blood samples were collected from each participant by trained medical staff. All subjects completed a questionnaire that asked for the worker's medical history and family clinical history, and 195 of them (96.5%) also completed a self-administered detailed questionnaire about lifestyle habits, mental stress, occupational status, etc. Subjects who received medication for diabetes, hypertension, or cancer were excluded from the study. This study was approved by the Ethics Committee at Osaka University School of Medicine, and written informed consent was obtained from all subjects.

### Assessment of anthropometrical data

The height (without shoes) of each subject was measured in centimeters, and weight (without shoes and in light clothing) was measured in kilograms (TANITA). Body mass index (BMI) was calculated as weight in kilograms over height in meters squared.

Blood pressure was measured, by trained nurses using a digital blood-pressure monitor (Inteli Sense, HEM-907, OMRON) on the right arm, twice with the subject in the sitting position and with at least 5 min rest between the two measurements. The values used in this study were the average of the two measurements.

### Evaluation of lifestyle factors and medical history

Lifestyle habits were assessed by using a self-administered questionnaire that asked about physical activity, habitual dietary intake, alcohol drinking habits, and smoking. The physical activity of the subjects was assessed by asking them about physical exercise, hours of walking on weekdays, sleeping hours, and hours of TV-watching, work, and physical activity in leisure time. Habitual dietary intake was assessed by asking about the usual average consumption of 15 foods and about 5 behaviors: the frequency of eating out, the meal for weigh loss, whether they ate within the 2 hours before bedtime, whether they eat too much, whether they eat too fast. The frequency of food consumption was queried using four categories: everyday, often, sometimes, and never. Alcohol drinking habits were assessed by ask about drinking frequency and alcohol consumption per occasion. Subjects were asked about smoking status and were classified into three categories: current smoker, ex-smoker, and nonsmoker. Current smokers and ex-smokers were asked about the number of cigarettes smoked each day and about how many years they had been smoking.

Public health nurses questioned all 202 of the subjects about their medical history and their family clinical history.

### Measurement of Biochemical Variables

All blood data except leptin and adiponectin levels were measured in one laboratory (Shionogi Institute for Medical Science, Japan). Within two hours after blood samples for adiponectin and leptin were obtained, they were centrifuged at 3000 rpm for 25 min at -4°C before being stored at -80°C until the levels of the two adipocytekines were assayed. The laboratory-measured values of the serum lipids came from the Cholesterol Reference Method Laboratory Network, and the standardization values came from the Center for Disease Control and the Prevention/Cholesterol Reference Method Laboratory Network [28].

Plasma adiponectin concentration was determined in duplicate with an ELIZA assay (Otsuka Assay Institute, Japan) [2].

### Statistical Analysis

Plasma adiponectin concentrations were classified into two categories: <4.0 μg/ml (hypoadiponectinemia) and ≥4.0 μg/ml [12,19]. The statistics listed in Table 1 that described adiponectin levels, clinical characteristics, and lifestyle habits are means ± the standard deviation (SD). The chi-square test was used to compare dichotomous variables, and t testing was used to compare means between the two groups classified according to adiponectin level. Associations between adiponectin levels and clinical factors in these two groups were examined by using an age-adjusted partial correlation coefficient.

**Table 1:** Clinical Characteristics of 202 Japanese men by adiponectin levels

		**Adiponectin Levels**		
		
	**All**	**< 4.0 μg/mL**	**≥ 4.0 μg/mL**	P*
**N**	**202**	**77**	**125**	
Adiponectin (μg/mL)				
Mean	4.9 ± 2.2	2.9 ± 0.8	6.1 ± 1.9	.000
Median (Min, Max)	4.5 (0.58, 15.30)	3.0 (0.58, 3.96)	5.7 (4.02, 15.30)	.000
Age (years)	42.0 ± 10.3	42.3 ± 9.1	41.7 ± 11.0	.670
BMI (kg/m^2^)	23.6 ± 2.8	24.3 ± 2.9	23.2 ± 2.6	.005
SBP (mmHg)	126.0 ± 14.2	128.8 ± 16.2	124.2 ± 12.5	.035
DBP (mmHg)	75.7 ± 12.0	78.9 ± 13.4	73.7 ± 10.5	.004
Total chol. (mg/dL)	203.6 ± 36.0	212.1 ± 37.1	198.4 ± 34.5	.008
HDL chol. (mg/dL)	59.9 ± 14.2	55.3 ± 11.9	63.8 ± 14.7	.000
LDL chol. (mg/dL)	134.0 ± 34.2	139.5 ± 33.3	129.9 ± 34.5	.053
FBG (mg/dL) ^†^	89.7 ± 13.1	92.5 ± 16.9	88.0 ± 9.6	.000
Platelet (× 10^4 ^cells/μL)	23.6 ± 5.0	25.0 ± 5.5	22.6 ± 4.5	.002

Data are shown in mean ± SD.

* t-test

†log transformed

BMI, body mass index; SBP, systolic blood pressure; DBP, diastolic blood pressure; HDL, high-density lipoprotein; LDL, low-density lipoprotein; FBG, Fasting plasma glucose

Multiple logistic regression analysis models were used to evaluate the relations between hypoadiponectinemia and lifestyle habits. The dependent variable was the presence or absence of hypoadiponectinemia (<4.0 μg/ml), and the independent variables were four lifestyles habits: eating many deep-yellow vegetables, frequency of eating out, physical exercise, and smoking. In our model we adjusted for age, BMI, total cholesterol level, high-density lipoprotein (HDL) cholesterol level, hypertension, hyperglycemia, and family clinical history. Age was classified into four categories: 20–29 years, 30–39 years, 40–49 years, and 50–59 years. The subjects were divided into three categories according to BMI (≤20 kg/m^2^, 20.1–24.9 kg/m^2^, ≥25 kg/m^2^) and total cholesterol level (<160 mg/dl, 160–219.9 mg/dl, ≥220 mg/dl) and into two categories according to the presence or absence of hypertension (systolic blood pressure ≥130 mmHg and/or diastolic blood pressure ≥85 mmHg), low HDL cholesterol levels (<40 mg/dl), and hyperglycemia (a fasting blood glucose level ≥110 mg/dl). The total numbers of family members with a clinical history of diabetes mellitus, hypertension, stroke, heart disease, gout, or cancer (yes = 1, no = 0) were divided into four categories: 0, 1, 2, and 3+. All p values presented are two-tailed, and p <0.05 was considered statistically significant. Statistical analyses were performed using the statistical software SPSS version 11.5 (Texas Instruments, Chicago, IL) [29]

## Results

Statistics describing the clinical characteristics and adiponectin levels of the hypoadiponectinemic and normoadiponectinemic groups are listed in Table 1. The hypoadiponectinemia group had significantly higher levels of BMI, systolic blood pressure (SBP), diastolic blood pressure (DBP), total cholesterol, fasting blood glucose (FBG) and platelets and had significantly lower levels of HDL cholesterol. The number of family members with a clinical history was also greater in the hypoadiponectinemia group (1.25 ± 1.39 points /6 points) than in the normoadiponectinemic group (1.0 ± 1.03 points/6 points) (p < 0.18, t-test).

The partial correlation of adiponectin levels with selected anthropometric and biochemical factors are shown in Table 2. After adjustment for age, adiponectin levels were negatively correlated with BMI, SBP, DBP, total cholesterol, FBG, and platelet level and were positively correlated with HDL cholesterol in all participants. After adjustment for age and BMI, adiponectin levels were significantly correlated with lipid and platelet levels and trended to associate with DBP and FBG.

**Table 2:** Partial correlation of plasma adiponectin with anthropometric and biochemical factors (n = 202)

	***r*(Age-adjusted)**	***p***	***r*(Age,BMI-adjusted)**	***p***
BMI	-0.29	**		
SBP	-0.16	*	-0.05	0.46
DBP	-0.22	**	-0.11	0.11
Total chol.	-0.27	**	-0.20	*
HDL chol.	0.34	**	0.30	**
FBG^†^	-0.19	*	-0.13	*
Platelet	-0.18	*	-0.17	0.07

*p < 0.05, **p < 0.01

†:log transformed

Relations between lifestyle habits and plasma adiponectin levels can be seen in Table 3. Fewer than one fifth of the subjects who filled out the lifestyle questionnaire exercised at least twice a week, but the number of them that were hypoadiponectinemic was significantly smaller than the number that were normoadiponectinemic (2.1% versus 15.4% of the subjects who filled out the lifestyle questionnaire). The hypoadiponectinemic current smokers were 18.5% of the subjects who filled out the lifestyle questionnaire and the normoadiponectemic ex-smokers and nonsmokers were 39.0% of the subjects who filled out the lifestyle questionnaire. Eating many deep-yellow vegetables (p < 0.075) and the frequency of eating out (p < 0.053) trended to be associated with adiponectin levels but alcohol drinking habits did not. The results listed in Table 3 thus indicate that smoking, eating many deep-yellow vegetables, eating out frequently, and getting physical exercise are hypoadiponectinemia-related lifestyle factors.

**Table 3:** Characteristics of Lifestyle habits by adiponectin levels (n = 195)

		**Adiponectin**		
		
		**<4.0 μg/mL**	**≥4.0 μg/mL**	P*
	**N (%)**	**N (%)**	**N (%)**	
**Physical exercise**				
≥ Twice /week	34 (17.4)	4 (2.1)	30 (15.4)	.000
**Hours of walking in weekdays**				
≥30 hr/day	103 (53.1)	35 (18.0)	68 (35.1)	.373
**Smoking**				
Nonsmoker, Ex-smoker	113 (57.9)	37 (19.0)	76 (39.0)	.134
Current smoker	82 (42.1)	36 (18.5)	46 (23.6)	
**Alcohol drinking**				
None, Seldom	67 (34.4)	23 (11.8)	44 (22.6)	.537
**Many deep-yellow vegetables**				
Everyday	25 (12.8)	5 (2.6)	20 (10.3)	.075
**Many snack**				
Everyday	113 (57.9)	38 (19.5)	75 (38.5)	.231
**A lot of meat**				
Everyday, often	40 (20.5)	13 (6.7)	27(13.8)	.583
**Many deep-fry**				
Everyday, often	49 (25.1)	15 (7.7)	34 (17.4)	.307
**Many salty foods**				
Everyday, often	27 (13.8)	9 (4.6)	18 (9.2)	.675
**Frequency of eating out**				
< once a day	109 (55.9)	34 (17.4)	75 (38.5)	.053
**Sleeping hours**				
7 to 8 hr/day	47 (24.1)	14 (7.2)	33 (16.9)	.231

* χ^2 ^– test

We next used multiple logistic regression analysis to examine the association between hypoadiponectinemia and these four hypoadiponectinemia -related lifestyle factors (Table 4). After adjustment for multiple variables (age, BMI, hypertension, total cholesterol, HDL cholesterol, hyperglycemia, platelet level, and the number of family members with a clinical history), the risk of hypoadiponectinemia was significantly decreased by frequent physical exercise (= twice a week; Odds ratio [OR] = 0.21, 95% confidence interval [CI]= 0.06–0.74) and the frequent eating of many deep-yellow vegetables (everyday; OR = 0.25, 95%CI = 0.07–0.91) and was significantly increased by smoking (current smoker; OR = 2.08, 95% CI = 1.01–4.30) and by eating out frequently (=once a day; OR = 2.45, 95% CI = 1.19–5.08).

**Table 4:** Results of multiple logistic regression analysis of the association between hypoadiponectinemia and various lifestyle factors (n = 195). Dependent variable: hypoadiponectinemia (adiponectin <4.0 μg/ml = 1, ≥4.0 μg/ml = 0)

**Variables**	**OR**	**95% CI**	***p***
**Physical exercise**	0.21	(0.06 – 0.74)	0.015
(twice a week or more often = 1; once a week, a few times a month, never = 0)			
**Smoking**	2.08	(1.01 – 4.30)	0.047
(smoker = 1; nonsmoker, ex-smoker = 0)			
**Many deep-yellow vegetables**	0.25	(0.07 – 0.91)	0.035
(everyday = 1; often, sometimes, never = 0)			
**Frequency of eating out**	2.45	(1.19 – 5.08)	0.016
(3 times a day, once a day = 1; a few days a week, a few days a month, never = 0)			

OR: odds ratio, 95% CI: 95% confidence interval

Adjusted for age, BMI, hypertension, total cholesterol, HDL cholesterol, hyperglycemia, platelet count, and the number of family history (diabetes mellitus, gout, stroke, hypertension, heart disease, or cancer).

## Discussion

We showed that that, in general Japanese men, eating out once a day or more and smoking are independently associated with a higher risk of hypoadiponectinemia, whereas getting physical exercise at least twice a week and eating many deep-yellow vegetables daily are significantly associated with higher adiponectin concentrations. We suggest that physical activity, dietary factors, and smoking are independently related to plasma adiponectin levels in general Japanese men. This is consistent with the results of a few earlier studies indicating that lifestyle factors may modulate adiponectin levels in the general male population.

We found that smoking is independently associated with hypoadiponectinemia in general Japanese men. Other investigators have reported that smoking is associated with adiponectin levels in healthy Japanese men as well as in Japanese men with hypertension, diabetes, and hyperlipidemia and that adiponectin levels are significantly lower in smokers after multiple adjustment. Furthermore, oxidative stress and nicotine reduce the expression and secretion of adiponectin in cultured mouse 3T3-L1 adipocytes [27]. Many studies also suggest that smoking induces inflammatory factors (TNF-α, CRP, IL-6, fibrinogen, etc.) that are risk factors for atherosclerosis and cardiovascular diseases [30,31]. Adiponectin accumulates in the subendothelial space of injured vascular walls and inhibits the transformation of macrophages to foam cells [15,32]. Our findings and those of previous studies suggest that adiponectin levels are a useful biomarker for evaluating the effects of smoking on the risk of atherosclerosis and cardiovascular diseases in the general Japanese population.

Our results also showed that daily diets rich in deep-yellow vegetables are associated with a significantly lower risk of hypoadiponectinemia and that eating out once or more a day is associated with a significantly higher risk of hypoadiponectinemia. Some previous reports have suggested that dietary factors are related to adiponectin levels in human beings. They showed that a Mediterranean-style diet and a high-fiber diet with a low caloric and low glycemic load were associated with higher adiponectin levels. Esposito et al. reported that a weight-loss program that included exercise increased the plasma adiponectin levels of obese women in a randomized trial [22]. They also showed that lower adiponectin levels were associated with diets including whole-grain products, legumes, fruits, vegetables, fish, and olive oil. Pischon et al. found in a cross-sectional study of 532 men without a history of cardiovascular disease that a diet with a high glycemic load was significantly associated with lower adiponectin levels that and carbohydrate intake tended to be associated with lower adiponectin levels [25]. Qi et al. showed in a cross-sectional analysis of 780 diabetic patients that diets low in glycemic load and high in fiber might increase plasma adiponectin concentrations [24]. A higher frequency of eating out has also been found to be associated with adverse nutritional consequences related to increased obesity [33]. Dietary factors are closely related to obesity and the development of type 2 diabetes and cardiovascular disease. We suggest that dietary factors independently modulate adiponectin levels in general Japanese men and that improving dietary factors can increase adiponectin levels and thereby reduce the risk of developing type 2 diabetes and cardiovascular disease.

We observed an independent association of more frequent physical exercise with higher adiponectin levels in general Japanese men. Adiponectin stimulates glucose utilization and fatty-acid oxidation by activating AMP kinase in muscle and enhances insulin action [34,35]. Exercise also improves glucose utilization and fatty-acid oxidation by activating AMP kinase in muscle [36]. Previous studies have evaluated small samples of the participants in exercise programs or weight-loss programs for the short term, and this has led to variability in results showing a relation between adiponectin and exercise [37-41]. Yokoyama et al. reported that aerobic exercise might increase plasma adiponectin levels in diabetes subjects when an intervention is accompanied by a reduction in weight or fat mass, but that study evaluated only 40 subjects and used only a three-week intervention [41]. The results of some studies with longer interventions suggested that regular physical activity and exercise in a weight-loss program increase adiponectin levels [22]. And previous epidemiological studies have shown that high levels of physical activity independently improved IL-6, C-reactive protein, leptin, TNF-α, and fibrinogen in healthy individuals [42,43]. We showed an independent association between exercise and adiponectin in general Japanese men. Our results suggested that regular exercise independently increased adiponectin levels.

Our study had some limitations. The cross-sectional design limited causal inferences. Although 71.2% of the hypoadiponectinemia subjects in our study did not change their diet habits and exercise within the last six months (chi-square test X^2 ^= 4.36; df = 1 and p = 0.037), many cohort studies in the future should explain the association of adiponectin and lifestyle in Japanese men and women. Our assessment of dietary factors was based on self-reported dietary intake and questionnaires that asked about some dietary habits and the simple frequency of food factors. Although we thus did not evaluate dietary factors in detail, our results linking dietary factors and adiponectin levels were similar to those of previous studies. Moderate alcohol intake was not independently associated with higher adiponectin levels in our study. No report has explained the mechanisms that associate moderate alcohol intake and adiponectin expression, but Pischon et al. showed moderate alcohol consumption was independently associated with higher adiponectin levels in men living in the United States [25,26]. It is necessary to further study the association of adiponectin levels with alcohol consumption in the Japanese population.

In conclusion, our findings showed that adiponectin levels in general Japanese men are independently modulated smoking, dietary factors, and physical exercise. We have suggested that lifestyle habits might independently modulate adiponectin levels and that adiponectin might be the useful biomarker helping people prevent type 2 diabetes and cardiovascular disease by modifying their lifestyles.
